# HPV38 impairs UV-induced transcriptional activation of the IL-18 pro-inflammatory cytokine

**DOI:** 10.1128/msphere.00450-23

**Published:** 2023-10-25

**Authors:** Maria Grazia Ceraolo, Maria Carmen Romero-Medina, Simone Gobbato, Giusi Melita, Hanna Krynska, Cecilia Sirand, Purnima Gupta, Daniele Viarisio, Alexis Robitaille, Jacqueline Marvel, Massimo Tommasino, Assunta Venuti, Tarik Gheit

**Affiliations:** 1 International Agency for Research on Cancer (IARC), World Health Organization, Lyon, France; 2 Biotechnology and Cell Signaling (CNRS/Université de Strasbourg, UMR 7242), Ecole Superieure de Biotechnologie de Strasbourg, Boulevard Sébastien Brant, Illkirch, France; 3 German Cancer Research Center, Heidelberg, Germany; 4 CIRI, Centre International de Recherche en Infectiologie, Université Lyon, Inserm U1111, Université Claude Bernard Lyon 1, CNRS, UMR5308, ENS de Lyon, Université Lyon, Lyon, France; 5 IRCCS Istituto Tumori Giovanni Paolo II of Bari, Bari, Italy; Northwestern University, Chicago, Illinois, USA

**Keywords:** HPV38, UV irradiation, p53, ΔNp73α, IL-18

## Abstract

Increasing evidence suggests that the beta genus of human papillomavirus (β-HPV) cooperates with ultraviolet B (UVB) radiation in inducing non-melanoma skin cancer (NMSC). For instance, expression of E6 and E7 oncoproteins from cutaneous human papillomavirus type 38 (HPV38) in a transgenic mouse model (Tg38) leads to the development of squamous cell carcinoma (SCC) after chronic UVB exposure. Transcriptomic analysis of the Tg38 SCC samples revealed a deregulation of inflammasome pathway-related genes such as IL-18, which is consistent with the well-known capability of several oncogenic viruses to modulate the immune response, in order to efficiently produce new progeny. However, the mechanism at the gene expression level by which UVB and HPV38 synergize and modulate the innate immune response has not been elucidated yet. Here, we propose a new molecular mechanism for the regulation of the IL-18 promoter that involves direct binding of p53 to specific response elements. This mechanism seems to be impaired in the presence of the β-HPV38 oncoproteins. Instead, a newly described inhibitory complex formed by DNA methyltransferase 1 (DNMT1)/PKR/ΔNp73α is recruited to the region formerly occupied by p53 in primary keratinocytes. Together, these results corroborate our hypothesis that β-HPV38 plays an important role in the inhibition of the ultraviolet-induced inflammasome response. This downregulation is likely to impair the ability of keratinocytes to recruit the immune cell population at the UVB-exposed area, causing a defect in the clearance of cells harboring DNA damage and promoting a favorable environment for the progression of NMSC.

**IMPORTANCE:**

Here, we demonstrate that the direct binding of p53 on the IL-18 promoter region regulates its gene expression. However, the presence of E6 and E7 from human papillomavirus type 38 impairs this mechanism via a new inhibitory complex formed by DNA methyltransferase 1 (DNMT1)/PKR/ΔNp73α, which binds to the region formerly occupied by p53 in primary keratinocytes.

## INTRODUCTION

The most common type of malignancy in adult Caucasian populations is non-melanoma skin cancer (NMSC), which accounts for more than 3 million cases annually in the United States alone (1).

The etiology of NMSC is complex because it may be caused by a combination of environmental, phenotypic, or genotypic factors (2).

Several studies have reported that exposure to ultraviolet B (UVB) radiation is a key risk factor in the development of skin cancer. In addition, immunocompromised individuals, such as solid-organ transplant recipients and HIV-positive individuals, are more prone to develop NMSC compared with the general population (3–5).

The importance of host immune status strongly supports a potential role of opportunistic infectious agents in these events (6). In particular, cutaneous beta human papillomavirus (β-HPV) is considered the most likely additional etiological factor for NMSC, acting as a co-risk factor together with ultraviolet (UV) radiation. This association was first described in patients with epidermodysplasia verruciformis, a skin disease caused by an autosomal recessive disorder in the TMC6 (EVER1) and TMC8 (EVER2) genes. Those individuals are more susceptible to β-HPV infections and to the development of cutaneous squamous cell carcinoma (cSCC) after sunlight exposure (7, 8).

Functional studies, performed in experimental models both *in vivo* and *in vitro*, have highlighted the oncogenic properties of E6 and E7 proteins of some β-HPV types (9, 10) and their ability to evade the host immune response (11, 12) in order to establish a productive infection.

A transgenic human papillomavirus type 38 (HPV38) mouse model (Tg38), which expresses E6 and E7 of β-HPV38 in the skin under the control of the keratinocyte K14 promoter (K14 HPV38 E6/E7), did not show any spontaneous formation of cancer lesions under physiological conditions, whereas after UV exposure, Tg38 mice developed actinic keratosis-like lesions, which are considered a precursor of cSCC in humans. In contrast, no skin lesions were observed in wild-type mice after UV irradiation (13, 14).

Independent studies carried out on keratinocyte cell lines provided evidence that exposure to UV radiation leads to the activation of pathways involved in the innate immune response, such as the inflammasome, with the subsequent secretion of specific pro-inflammatory cytokines such as IL-1β or IL-18 (15). However, E6 and E7 from α-HPV16 as well as β-HPV38 abrogated the secretion of the pro-inflammatory cytokine IL-1β after UVB exposure (16).

In addition, the analysis of the transcriptome profile of Tg38 mice showed an impairment of inflammasome pathways after UVB irradiation, leading to reduced IL-18 mRNA production, by an unknown mechanism (17). IL-18 is an immunoregulatory cytokine that is constitutively expressed in human keratinocytes. Upon processing and release, IL-18 induces IFNγ production in NK cells and Th1 polarization of CD4+ T cells (18, 19), contributing to the immune anti-viral response (20). IL-18 is also involved in DNA repair after UV irradiation (21, 22) as well as in cancer development or suppression (23, 24).

It is also well known that UVB exposure of keratinocytes induces an accumulation of the tumor suppressor gene p53 (25, 26) and that its expression is altered by β-HPV38 (27). As shown by Accardi et al., E7 oncoprotein from β-HPV38 cooperates with E6 to induce p53 stabilization and accumulation, which leads to the transcription of ΔNp73α, an isoform of p73. Furthermore, E7 directly regulates IKKβ nuclear translocation and promotes the formation of the complex ΔNp73α/IKKβ/DNA methyltransferase 1 (DNMT1)/EZH2, which binds different p53-related promoters, in order to inhibit their activation (28, 29). Interestingly, previous studies have shown that p53 upregulates components of the innate immune response, such as the TLR9 receptor, after UV irradiation in keratinocytes. However, p53-mediated upregulation is inhibited in cells expressing HPV38 oncoproteins (30).

Together, these data suggest that β-HPV38 alters the cellular response to UVB radiation at multiple levels, such as the regulation of inflammasome-dependent cytokines as well as the pathways involved in DNA repair. However, regardless of the evidence of this cooperation, the mechanism through which β-HPV38 and UV radiation interact to cause NMSC has not been fully elucidated yet. Here, we aim to further dissect the mechanisms of this process by highlighting a novel molecular mechanism for the regulation of the IL-18 promoter that involves p53. Our data suggest that p53 acts directly on the IL-18 promoter by binding to specific sites and promoting the transcription of the cytokine. This mechanism seems to be impaired in the presence of β-HPV38 E6 and E7 oncoproteins, where p53 induces ΔNp73α expression, which ultimately occupies the promoter and inhibits IL-18 gene expression. Hence, UVB-induced p53 accumulation in the presence of HPV38 could promote anti-viral immune escape while promoting a favorable environment for cancer progression via the indirect modulation of the inflammasome pathway.

## RESULTS

### HPV38 inhibits IL-18 gene expression

Epidemiological and biological findings indicate that cutaneous β-HPV and UV radiation synergize in promoting skin carcinogenesis (17).

**Fig 1:**
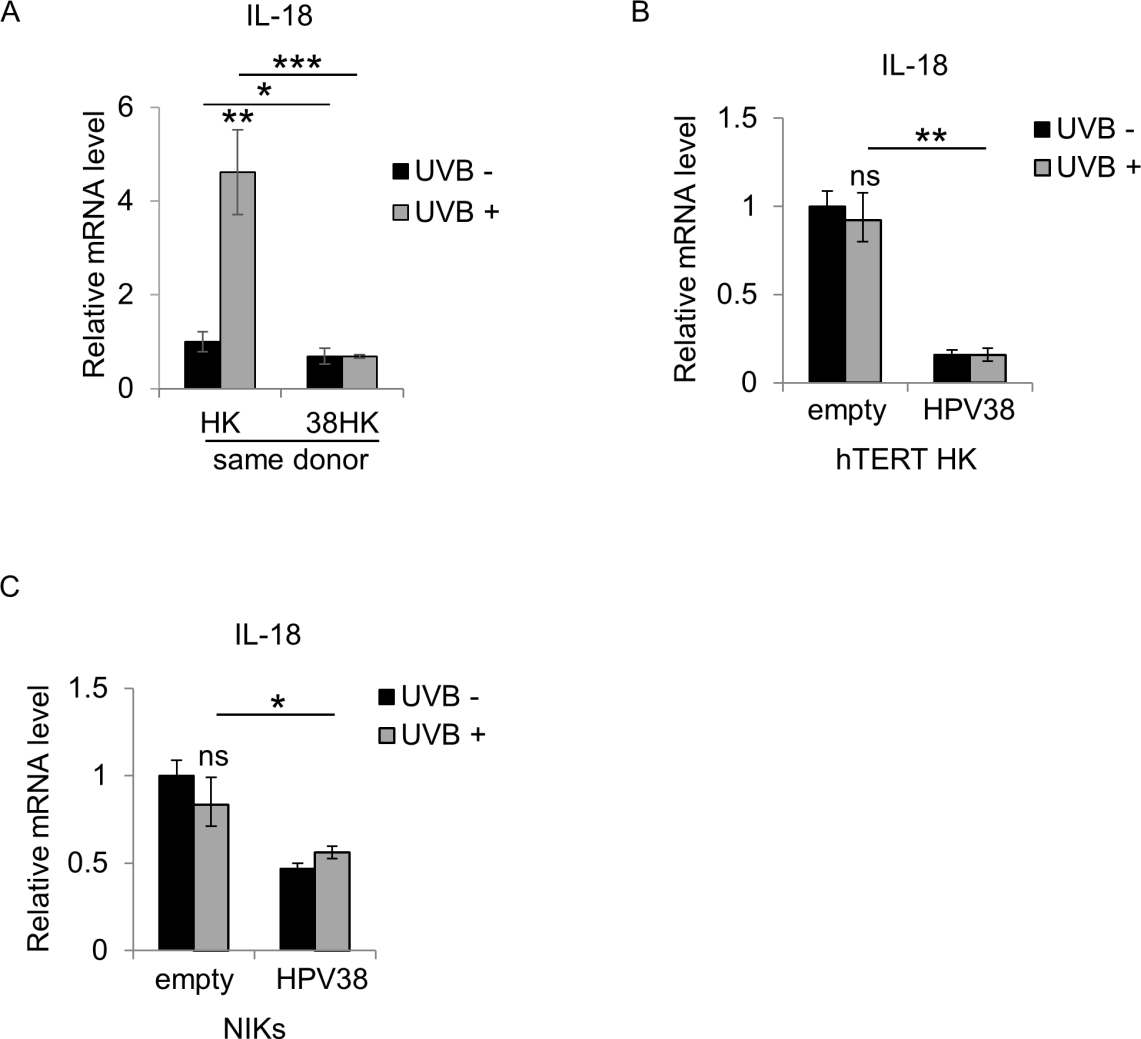
IL-18 gene expression is impaired in HPV38 E6/E7-expressing cells. (**A–C**) Total RNA was extracted from UVB-irradiated and mock-irradiated HK or 38HK (**A**), HK expressing the human telomerase reverse transcriptase (hTERT) gene (**B**) and the NIKs cell line (**C**). IL-18 mRNA levels were measured by RT-qPCR and normalized to GAPDH. Data are means of three independent experiments performed in two different donors. *P < 0.05; **P < 0.01; ***P < 0.001; ns, not significant.

To evaluate the impact of the viral proteins on the UV-induced transcriptional activation of IL-18, we used human primary keratinocytes, expressing (38HK) or not expressing (HK) E6 and E7 from HPV38, as an experimental model. Cells were irradiated with a dose of UVB (50 mJ/cm^2^) and collected after 8 h. In agreement with previous published work (15), real-time quantitative PCR (RT-qPCR) showed an upregulation of IL-18 mRNA levels in HK cells, which was strongly downregulated in the presence of E6 and E7 from HPV38 (Fig. 1A).

To further confirm our findings, we developed HK expressing the human telomerase reverse transcriptase (hTERT) gene, to prolong the life span of the primary cells. In addition, we used alternative models such as human spontaneously immortalized keratinocytes, i.e., the NIKs cell line. All the cell lines were stably transduced with a retrovirus expressing the two HPV38 oncogenes, E6 and E7. Cells were irradiated with a dose of UVB (50 mJ/cm^2^) and collected after 8 h. Surprisingly, RT-qPCR revealed that none of these cell lines were able to upregulate IL-18 mRNA expression when exposed to UVB radiation. However, the expression of the two viral oncoproteins downregulated the basal IL-18 mRNA levels (Fig. 1B and C). These results highlighted the loss of IL-18 transcriptional activation after UVB radiation in immortalized keratinocytes (i.e., hTERT-HK and NIKs) compared with primary cells (HK), thus suggesting that, similarly to E6 and E7 from HPV38, the immortalization alters the transcriptional functions of potential transcription factors involved in the UVB-induced IL-18 gene expression.

### p53 is a key player in IL-18 gene expression and is the target of HPV38-mediated inhibition

**Fig 2:**
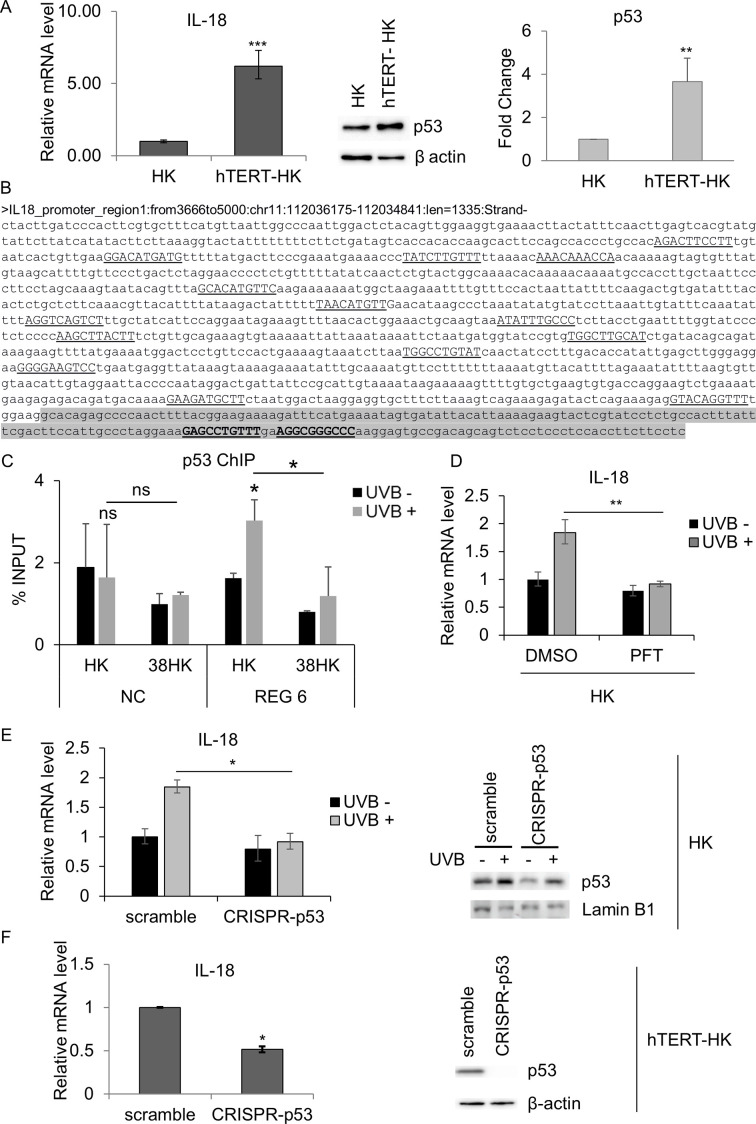
UV-induced IL-18 transcriptional activation is mediated by p53 recruitment to a specific region of the IL-18 promoter. (**A**) IL-18 mRNA levels were measured by RT-qPCR and normalized to GAPDH (left panel). p53 protein levels were evaluated by immunoblotting (IB). β-Actin was used as a loading control. The amounts of total p53 were quantified using Image Lab Software (Bio-Rad). After normalization of the levels of total p53 to the signal for β-actin, the relative level of p53 in hTERT-HK was evaluated compared with primary keratinocytes (HK). Images shown are examples of three independent experiments (right panel). Results shown are the means of data from three independent experiments. ***P ≤ 0.001; **P ≤ 0.01. (**B**) Promoter region of the human IL-18 gene. The genomic coordinates are relative to the human genome build hg19. The putative p53 response elements (REs) are underlined. p53 REs were predicted using TFBind software and the JASPAR database. Region 6 is highlighted. (**C**) Chromatin immunoprecipitation (ChIP) was performed on mock-irradiated (−) and UVB-irradiated (+) normal HK to evaluate p53 recruitment to the IL-18 promoter. Results were analyzed by qPCR with primers spanning the IL-18 region 6 or the intergenic region of chromosome 22 as a negative control (NC). The data shown are representative of three independent experiments. *P < 0.05. (**D**) HK were UVB irradiated (+) or mock irradiated (−) and then cultured in medium containing cyclic pifithrin-α hydrobromide (PFT) or DMSO as a control for 8 h. IL-18 mRNA levels were determined by RT-qPCR. GAPDH was used as an internal control for each reaction. Data shown are the means of three independent experiments. **P ≤ 0.01. (**E**) HK either expressing wild-type p53 (scramble) or with CRISPR/Cas9-mediated p53 deletion (CRISPR-p53) were UVB irradiated (+) or mock irradiated (−). IL-18 mRNA levels were determined by RT-qPCR. GAPDH was used as an internal control for each reaction. Data shown are the means of three independent experiments. *P ≤ 0.05 (left panel). p53 protein levels were evaluated by IB. Images shown are examples of three independent experiments (right panel). (**F**) IL-18 mRNA and p53 protein levels from hTERT-HK expressing wild-type p53 (scramble) or with CRISPR/Cas9-mediated p53 deletion (CRISPR-p53) were measured by RT-qPCR (left panel) and IB (right panel). Gene expression was normalized to GAPDH. Data shown are the means of three independent experiments. *P ≤ 0.05.

UVB radiation causes an accumulation of p53 in HK (25, 26). Moreover, it has been described that the ectopic expression of hTERT in HK cells induces p53 accumulation (31, 32). Accordingly, the analysis of IL-18 mRNA expression of the keratinocytes overexpressing hTERT (hTERT-HK) showed a strong upregulation of the basal IL-18 mRNA level compared with HK (Fig. 2A).

Therefore, we hypothesized that p53 could be involved in the induction of IL-18 in UV-exposed keratinocytes.

Based on this, we analyzed the IL-18 promoter region by using TFBind (PMID: 10487870) software and the JASPAR database (PMID: 34850907). The complete *in silico* analyzed region covered 5000 bp upstream of the IL-18 transcription start site (TSS), and the transcription factor (TF) prediction tools also revealed putative p53 TF binding sites in the region between 0 and 3,665 bp upstream of the TSS. Loci prediction of p53 TF binding sites reported by both tools was evaluated (TFBind similarity value ≥0.79 and JASPAR relative score ≥0.70). The analyses revealed the presence of at least 16 putative p53 response elements (REs) in a region upstream of the TSS (Fig. 2B).

To evaluate the involvement of p53 in the regulation of IL-18, we divided the promoter into six regions and designed six different pairs of primers in order to cover all the putative binding sites (Table 1). Next, we performed chromatin immunoprecipitation (ChIP) experiments in HK and 38HK exposed or not to UV radiation. The results showed a significant enrichment of p53 binding on specific REs located in region 6 in HK cells upon UVB irradiation. In contrast, HPV38 E6 and E7 prevented the recruitment of p53 to the IL-18 promoter (Fig. 2C).

**TABLE 1:** Oligonucleotide sequences for gene knockdown, RT-qPCR, and ChIP experiments^*a*^

Target	Sequence
Gene knockdown	
Scrambled RNA (negative control)	5′-GGUGGAAGAGGUGGUGAGC-3′
ΔNp73α sense	F: 5′-CCATgctgtacgTCGGt-3′
ΔNp73α antisense	F: 5′-ACCGacgtacagCATGg-3′
p53 vector #1	F: 5′-TCCATTGCTTGGGACGGCAAGTTTT-3′
	R: 5′-TTGCCGTCCCAAGCAATGGACGGTG-3′
p53 vector #2	F: 5′-CCATTGTTCAATATCGTCCGGTTTT-3′
	R: 5′-CGGACGATATTGAACAATGGCGGTG-3′
p53 vector #3	F: 5′-CTCGGATAAGATGCTGAGGAGTTTT-3′
	R: 5′-TCCTCAGCATCTTATCCGAGCGGTG-3′
p53 vector #4	F: 5′-CACTTTTCGACATAGTGTGGGTTTT-3′
	R: 5′-CCACACTATGTCGAAAAGTGCGGTG-3′
Scrambled vector	F: 5′-GGATGGACGGTAGAGGTGGGTTTT-3′
	R: 5′-CCACCTCTACCGTCCATCCCGGTG-3′
Primers for RT-qPCR	
IL-18	F: 5′-CTCGGATAAGATGCTGAGGAGTTTT-3′
	R: 5′-TCCTCAGCATCTTATCCGAGCGGTG-3′
ΔNp73α	F: 5′-AACCATGCTGTACGTCGGTGACCCC-3′
	R: 5′-GCGACATGGTGTCGAAGGTGG-3′
PKR	R: 5′-CAAGAGGTTTGGCATGGATT-3′
	R: 5′-GCTCCGCCTTCTCGTTATTA-3′
GAPDH	F: 5′-AAGGTGGTGAAGCAGGCGT-3′
	R: 5′-GAGGAGTGGGTGTCGCTGTT-3′
Primers for ChIP analysis	
Negative control region for ΔNp73α	F: 5′-CCGGAAGCACTTCTCCTAGA-3′
	R: 5′-AAGAGAGAGCGGAAGTGACG-3′
Chr22 (negative control)	F: 5′-GGTGCTCCTGGAAGCTGGGC-3′
	R: 5′-AAGGCAGCTGGCGTGAGGC-3′
p53 RE on IL-18 promoter region 6	F: 5′-GCACAGAGCCCCAACTTTTA-3′
	R: 5′-AAGAAGGTGGAGGGAGGAGA-3′

^*a*^ In ΔNp73α sense and antisense oligonucleotides, the capital letters indicate phosphorothioate nucleotides.

In line with this finding, the inhibition of p53 functions in primary cells, by using the chemical inhibitor pifithrin-α (PFT), blocked UV-mediated activation of IL-18 expression (Fig. 2D).

In addition, we generated p53 knockout HK cells using CRISPR/Cas9 technology. Accordingly, after UVB irradiation, the IL-18 mRNA levels were not upregulated when p53 expression was abrogated by the CRISPR/Cas9 vector compared with control scramble vector (Fig. 2E).

To further corroborate the link between p53 and IL-18 expression, we generated p53 knockout hTERT-HK using CRISPR/Cas9 technology. IL-18 mRNA expression was evaluated by RT-qPCR, which showed downregulation of IL-18 expression after p53 knockdown (Fig. 2F). These findings support our hypothesis that p53 may be involved in the regulation of IL-18 expression.

### ΔNp73α is involved in the inhibition of IL-18 expression in HPV38 keratinocytes

It has already been reported that HPV38 E6 and E7 expression induces the accumulation of ΔNp73α in 38HK, which acts as a dominant-negative form of p53 functions (27, 28).

**Fig 3:**
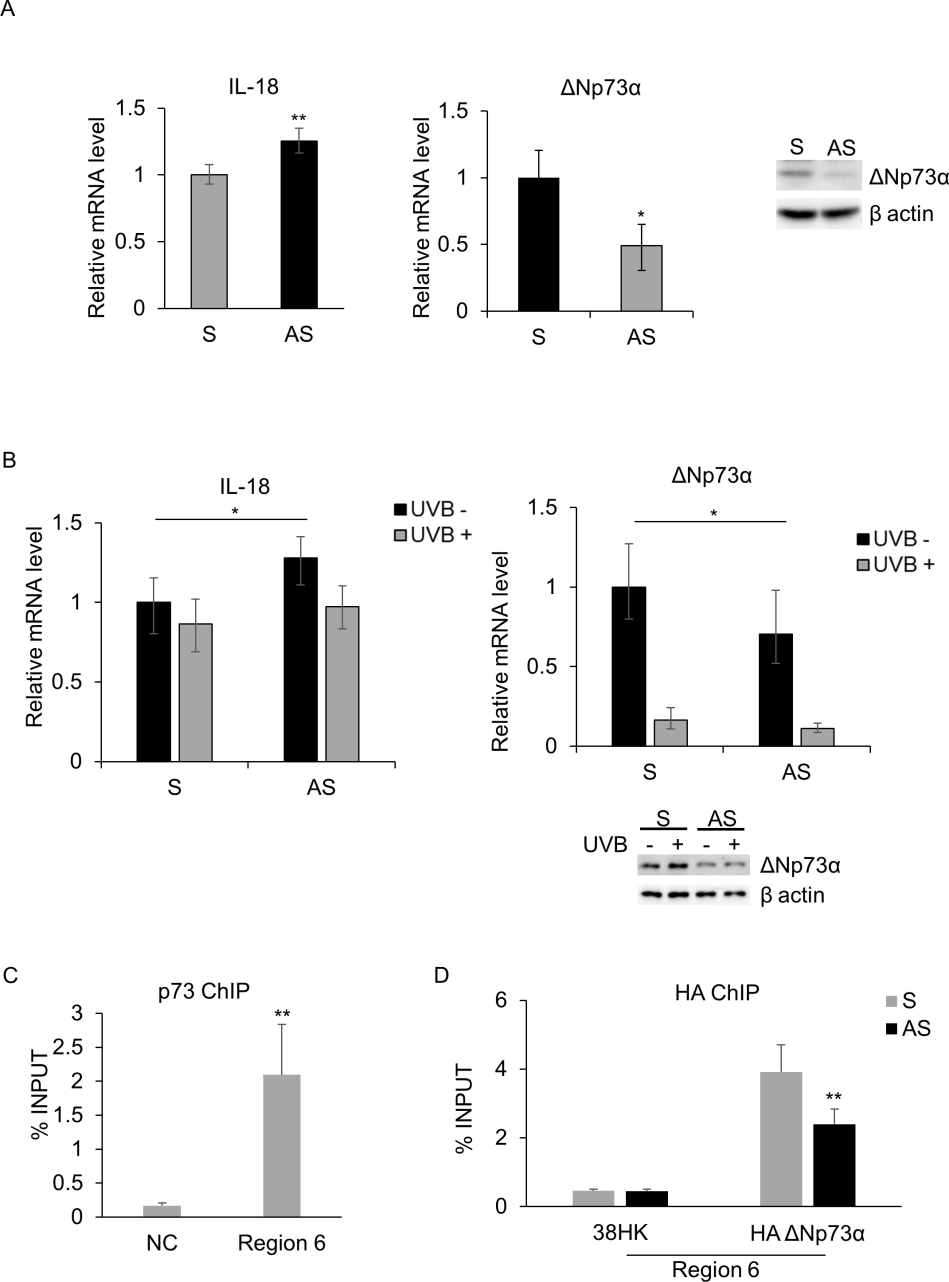
ΔNp73α downregulates IL-18 expression. (**A and B**) 38HK cells were transfected with sense (control, **S**) and antisense (AS) oligonucleotides against ΔNp73α. (**A**) After 24 h, cells were collected and processed for RT-qPCR (left) and IB (right). (**B**) After 24 h, cells were UVB irradiated (+) or mock irradiated (−) and collected after 8 h. RT-qPCR (left) and IB (right) were performed. (**C**) Chromatin from 38HK was processed for ChIP experiments using p73 antibody. Results were obtained by qPCR with primers spanning the IL-18 region 6 or a negative control region (NC). (**D**) ChIP assays using HA antibody were performed in 38HK transiently transfected with S (control) and AS oligonucleotides against ΔNp73α. (**A–D**) Results were obtained by qPCR. Error bars represent standard deviations from three independent experiments. *P ≤ 0.05; **P ≤ 0.01.

Therefore, we assessed whether ΔNp73α plays a role in the regulation of IL-18 expression by knocking down its expression. 38HK transfected with ΔNp73α antisense oligonucleotides showed a modest tendency to increase IL-18 mRNA levels compared with control cells (sense oligonucleotides) (Fig. 3A). Next, we evaluated the impact of UVB irradiation on IL-18 modulation in ΔNp73α-depleted 38HK.

No significant changes in IL-18 mRNA levels were detected in 38HK transfected with ΔNp73α antisense oligonucleotides (AS) and irradiated with a dose of UVB (50 mJ/cm^2^) (Fig. 3B). Although the magnitude of the IL-18 fold increase is modest for both experiments, it appears to be consistent and is statistically significant.

In addition, we performed a ChIP assay with an anti-p73 antibody, which showed an enrichment in region 6 compared with the negative control (Fig. 3C), indicating binding of p73 to this IL-18 promoter region.

To validate that ΔNp73α isoform was able to bind this promoter region, we carried out ChIP experiments in 38HK stably expressing ΔNp73α fused to an HA tag (HA-ΔNp73α). The cells were further transfected with ΔNp73α antisense or sense oligonucleotides and collected after 48 h. 38HK cells were used as a negative control.

The results showed a strong enrichment in region 6 when the ChIP was performed with the anti-HA antibody, indicating that the ΔNp73α isoform was binding to this promoter region. Accordingly, we reported a significant reduction in HA recruitment to region 6 from the IL-18 promoter when ΔNp73α was depleted with AS oligonucleotides (Fig. 3D).

### DNMT1 and PKR cooperate to regulate IL-18 expression in HPV38 keratinocytes

We have previously shown that ΔNp73α can form an inhibitory complex with specific epigenetic enzymes, such as DNMT1, which is known to be associated with gene expression silencing, in order to regulate different p53-related genes (28, 29).

Based on this, we investigated whether the ΔNp73α/DNMT1 complex could be involved in the downregulation of IL-18 levels in 38HK.

**Fig 4:**
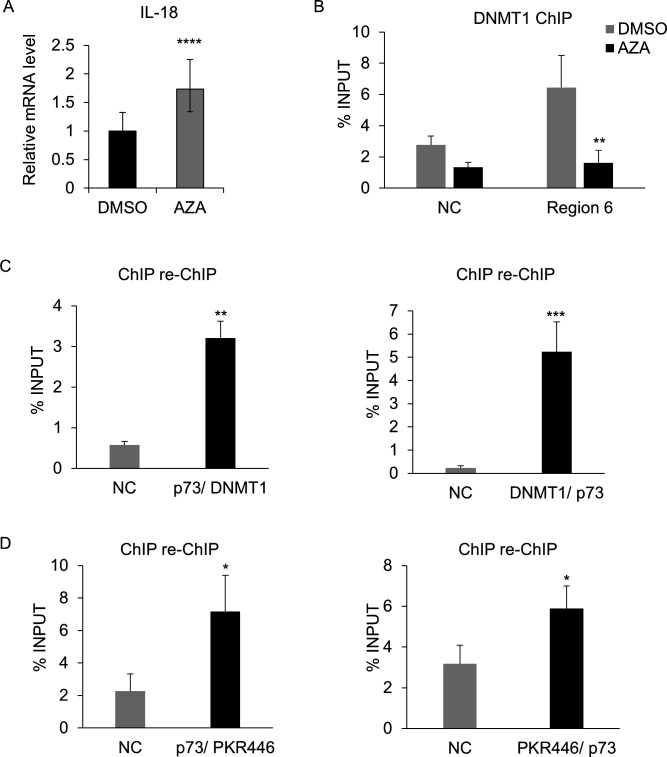
DNMT1 and PKR form an inhibitory complex on the IL-18 promoter in HPV38 keratinocytes. (**A**) IL-18 expression was evaluated by RT-qPCR after 24 h of treatment with 5-aza-2′-deoxycytidine (Aza) or DMSO at 30 µM final concentration. Error bars represent standard deviations from three independent experiments. ****P < 0.0001. (**B**) ChIP was performed on 38HK cells after treatment with Aza or DMSO as previously described. Results were obtained by qPCR using primers for the IL-18 promoter region 6. **P ≤ 0.01. (**C and D**) Chromatin was processed for a ChIP-reChIP assay in which p73-immunoprecipitated DNA was re-immunoprecipitated by DNMT1 (**C**) or PKR Thr446 (**D**), and vice versa. Enrichment of antibodies binding to the IL-18 region 6 or the intergenic region of chromosome 22 (NC) was obtained by qPCR. Data shown are the means of three independent experiments performed in triplicate. *P < 0.05; **P < 0.01; ***P < 0.001.

The inhibition of DNMT1 with 5-aza-2′-deoxycytidine (Aza), which is a 2′-deoxycytidine analogue and a global demethylating agent, resulted in a 2-fold increase in IL-18 mRNA expression in 38HK cells (Fig. 4A). This event corresponds to a loss of DNMT1 recruitment to the IL-18 promoter (Fig. 4B).

In addition, ChIP-reChIP experiments showed the interaction between ΔNp73α and DNMT1 and their recruitment to the IL-18 promoter (Fig. 4C).

Finally, 38HK activates the double-stranded RNA-dependent protein kinase (PKR) (33), which plays a relevant role in regulating inflammation throughout the innate immune response (34, 35). Intriguingly, ChIP-reChIP assays revealed PKR binding on the IL-18 promoter together with DNMT1 (Fig. 4D).

Together, these findings show that ΔNp73α forms a complex with DNMT1 and PKR in order to inhibit IL-18 expression in HPV38-expressing keratinocytes.

### PKR phosphorylation plays a key role in the inhibition of IL-18 expression in HPV38 keratinocytes

**Fig 5:**
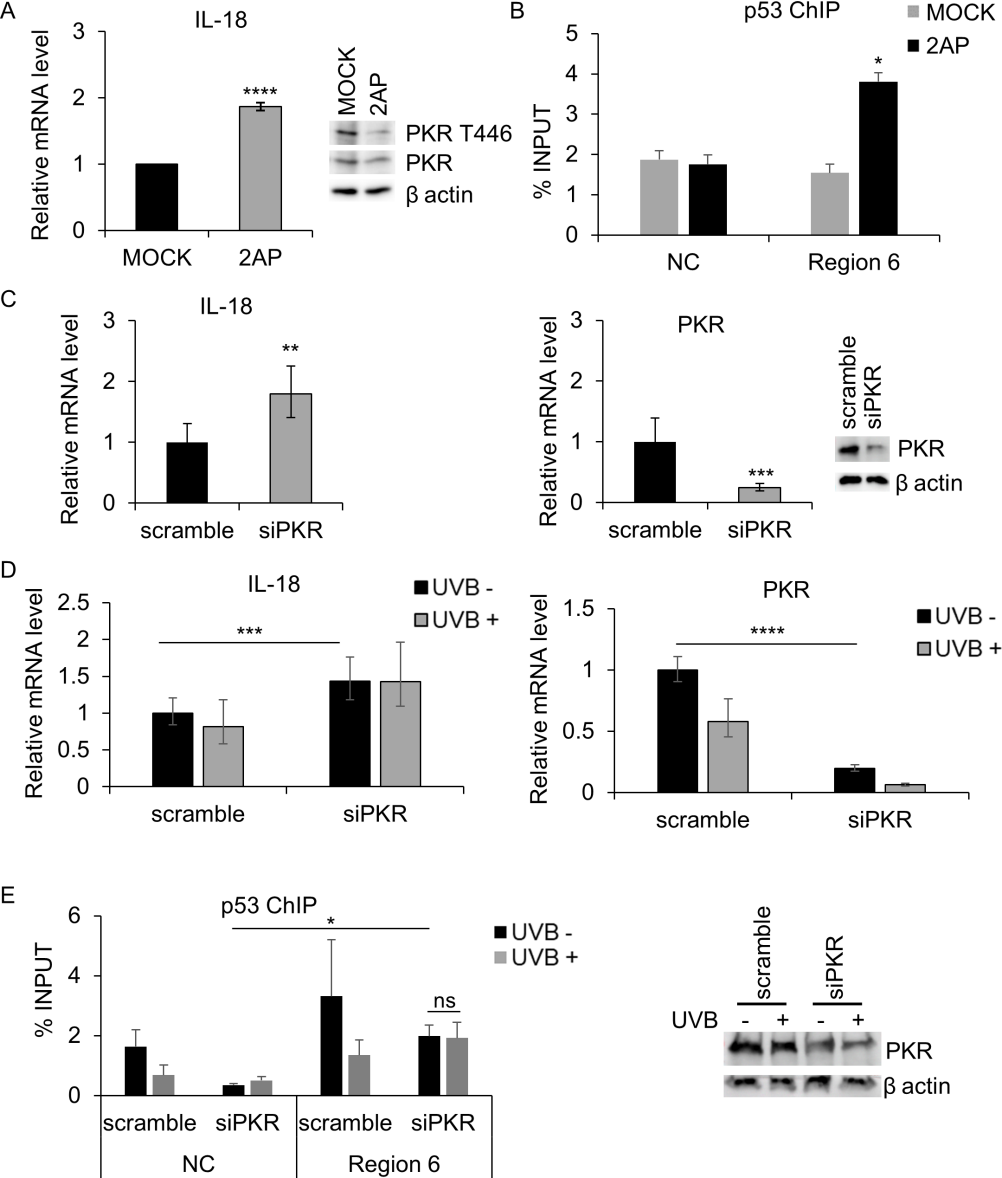
PKR phosphorylation on Thr446 is responsible for IL-18 inhibition. (**A**) 38HK cells were treated with the PKR inhibitor (2AP) or phosphate-buffered saline (PBS):glacial acetic acid (200:1) (MOCK) as a control. IL-18 expression was evaluated by RT-qPCR after 4 h of treatment. Protein extracts were analyzed by IB with the indicated antibodies (right panel). After incubation with phospho PKR T446 antibody, the membrane was stripped and incubated with total PKR antibody. Images shown are examples of three independent experiments. ****P < 0.0001. (**B**) ChIP was performed on 38HK cells after treatment with 2AP or PBS:glacial acetic acid, as previously described, using p53 antibody. Results were obtained by qPCR using primers for the IL-18 promoter region 6 and the intergenic region of chromosome 22 (NC). *P < 0.05. (**C and D**) 38HK were transiently transfected with PKR small interfering RNA pool (siPKR) or a relative control (scramble). After 24 h, the cells were harvested (**C**) or UVB irradiated (+) or mock irradiated (−) and collected after 8 h (**D**). IL-18 and PKR mRNA levels were measured by RT-qPCR and normalized to GAPDH. Data are means of three independent experiments. **P < 0.01; ***P < 0.001; ****P < 0.0001. Protein extracts were analyzed by IB with the indicated antibodies (C, right panel). (**E**) ChIP was performed in 38HK silenced for PKR (siPKR) or a relative control (scramble) and UVB irradiated (+) or mock irradiated (−). Cells were cross-linked, and chromatin was immunoprecipitated with p53 DO-1 antibody. *P < 0.05. The knockdown of PKR protein level was evaluated by IB (right panel).

To gain further insight into the regulation mechanism of IL-18 expression, we blocked PKR activity by treating 38HK cells with the chemical inhibitor 2-aminopurine (2AP). RT-qPCR showed a statistically significant increase in IL-18 mRNA levels (Fig. 5A).

Next, we hypothesized that in this scenario, the capacity of p53 to bind to the IL-18 promoter is rescued, as in primary keratinocytes.

ChIP assays confirmed that p53 is strongly recruited to the IL-18 promoter (Fig. 5B).

To corroborate these data, in parallel transient transfection experiments in 38HK, we observed that silencing PKR expression by using specific small interfering RNA (siRNA) statistically significantly increased IL-18 expression (2-fold, *P* ≤ 0.01) (Fig. 5C). IL-18 mRNA levels were not further affected in 38HK PKR-silenced and irradiated with a dose of UVB (50 mJ/cm^2^) (Fig. 5D). In addition, ChIP assays confirmed the recruitment of p53 in PKR-silenced cells, with a non-statistically significant change after UVB irradiation (Fig. 5E).

Altogether, these data suggest the inhibitory role of PKR on IL-18 expression by impairing p53 binding on the specific promoter region 6.

## DISCUSSION

The inflammasome is part of the innate immune system, and it is activated by different types of endogenous and exogenous stresses in several cell types (36). Previous studies have shown that UV radiation induces in human keratinocytes the activation of pathways involved in the innate immune response, such as the inflammasome complex (37). For instance, UVB irradiation triggers the cleavage and the subsequent release of specific pro-inflammatory cytokines, such as IL-1β or IL-18 (38, 39). Exposure to UVB radiation is one of the main risk factors leading to NMSC (40). Several lines of evidence support the hypothesis that β-HPV types, via the interaction of E6 and E7 viral proteins, promote the accumulation of UVB-induced DNA mutations, increasing susceptibility to skin carcinogenesis (13, 14, 41). However, the viral proteins appear to be dispensable after full development of SCC (14). In healthy skin, the activation of the inflammasome is a mechanism of protection against cancer development (42).

The role of the inflammasome in the development of cSCC is still unclear, aside from its role in allowing the release of IL-18 and the activation of the innate immune response. Schwarz and colleagues provided evidence for the involvement of IL-18 in DNA repair after UVB irradiation (21, 22). Hence, the inhibition of IL-18 gene expression by E6/E7 could participate in the transformation process. Here, we unravel a novel mechanism leading to the inhibition of IL-18 expression that could be involved in these events. By using primary keratinocytes as experimental models, we confirmed the upregulation of IL-18 expression when cells are exposed to UVB radiation, whereas the presence of HPV38 oncoproteins strongly reduced its expression. Interestingly, the experiments carried out in other cell lines, such as primary keratinocytes with an extended life span due to the overexpression of hTERT as well as NIKs, highlighted a loss in IL-18 mRNA transcriptional activation after UVB irradiation. However, the presence of the two viral oncoproteins downregulated the basal IL-18 mRNA expression levels in both cell lines. Of note, it is known that these cell lines show an altered status of p53 (31, 32, 43).

It has previously been described that p53 is stabilized after UVB exposure (25, 26). Moreover, keratinocytes expressing E6 and E7 HPV38 oncoproteins (HK38) also showed a stabilized wild-type form of p53, which selectively induced the expression of ΔNp73α, an isoform of the p53-related protein p73. The accumulation of ΔNp73α has been shown to be induced by other oncogenic viruses, such as Epstein–Barr virus (44) or human cytomegalovirus (45), which indicates a virus-dependent p53 inactivation (27, 28). Of note, in HK38 cells, p53 transcriptional function is antagonized by the p73 isoform ΔNp73α (27). Similarly to our work, several studies have described the contribution of p53 in the direct regulation of immune signaling pathways, i.e. TLR3, TLR9, or integrins, in response to cellular stimuli (30, 33, 46).

Of note, we identified a novel p53-mediated mechanism of IL-18 transcriptional activation in human primary keratinocytes. Our data showed that this recruitment of p53 to REs identified in the IL-18 promoter is impeded when HPV38 oncoproteins are present. In support of these findings, the inhibition of p53 functions in primary cells, by chemical inhibition or by knocking down its expression, resulted in a block of IL-18 expression after UV irradiation. Intriguingly, the ectopic expression of hTERT in primary keratinocytes induces an accumulation of p53 (31, 32) and thus, in turn, causes a strong upregulation of IL-18 mRNA level in the HK hTERT cell line compared with human primary keratinocytes. In addition, the deletion of the p53 gene by CRISPR/Cas9 technology in hTERT-HK corresponds to a reduction in basal IL-18 levels as well, thus reinforcing the hypothesis of a pivotal role of p53 in IL-18 promoter regulation. In addition, in a previous study, we demonstrated that ΔNp73α can be efficiently recruited to a specific binding site of TLR9, leading to a decrease in UV-induced p53 binding (30). Similarly, in this study, we showed that the silencing of ΔNp73α resulted in a slight but significant upregulation of the basal IL-18 mRNA levels; however, UV exposure did not further increase IL-18 activation. This event corresponds to binding of ΔNp73α to a specific site of the IL-18 promoter region in place of p53.

It is well known that epigenetic alterations (including DNA methylation and/or histone acetylation) can be influenced by many environmental factors, such as UV radiation and chemicals (47). In addition, several published data suggest a key role of DNA methylation in the regulation of inflammasome expression (48, 49). It has previously been reported that ΔNp73α forms an inhibitory complex with DNMT1 in HK38 (29, 33). In line with this finding, we demonstrated that inhibiting methylation, by using 5-aza-2′-deoxycytidine treatment, resulted in a modest upregulation of the basal IL-18 mRNA levels. In a complementary ChIP assay, we found that DNMT1 and ΔNp73α bind to the IL-18 promoter in the same region. The interaction between DNMT1 and ΔNp73α and their recruitment to the promoter was further confirmed by ChIP-reChIP experiments. These findings suggest that ΔNp73α can form an inhibitory complex that regulates IL-18 expression.

Interestingly, it has recently been demonstrated by our group that HPV38 oncoproteins strongly induce the accumulation of PKR, which leads to the phosphorylation of p53 at S392, thus promoting the formation of an inhibitory p53/DNMT1 complex on the integrin α-1 (ITGA-1) promoter (33). PKR plays a relevant role in anti-viral defense mechanisms and in inflammatory stress-mediated pathologies (50, 51). Also, several studies have highlighted the importance of PKR in the cytoplasm compartment, but only recently the focus has shifted to the potential role played by PKR in the nucleus (50). Thus, we hypothesized that DNMT1 could interact with PKR to regulate IL-18 response. Intriguingly, ChIP-reChIP assays performed in HK38 clearly showed the presence of the DNMT1/PKR/ΔNp73α complex in the region formerly occupied by p53 in primary keratinocytes. In addition, the inhibition of PKR, chemically or by gene silencing, led to the upregulation of IL-18 intracellular expression. This event is most likely due to the rescue of p53 binding on the IL-18 promoter. Our results suggest that the upregulation of IL-18 expression is related to the combined contribution of all three proteins composing the complex (ΔNp73α, DNMT1, and PKR) because knocking down the individual proteins yields only modest effects on IL-18 mRNA levels.

**Fig 6:**
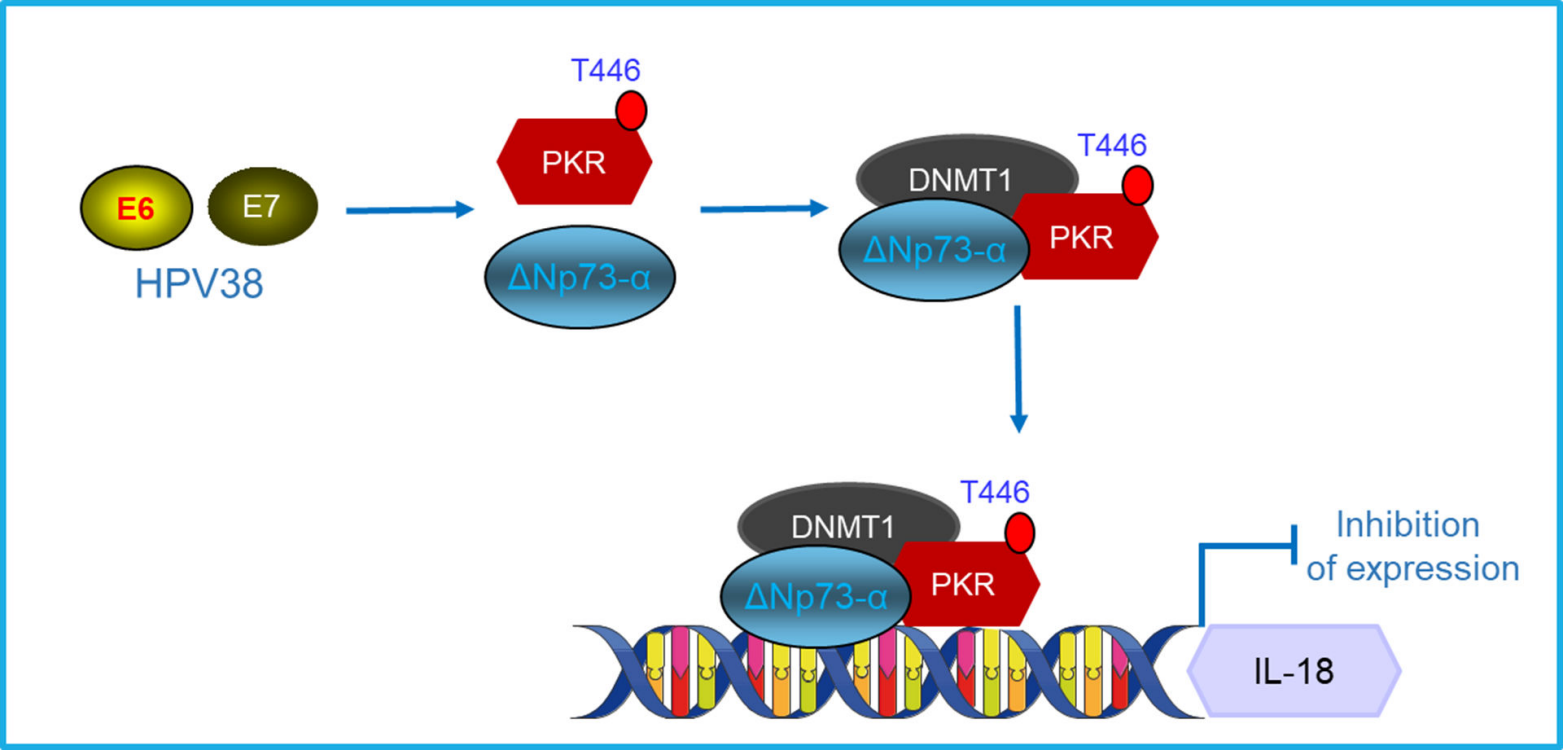
Proposed model for the HPV38-mediated impairment of the IL-18 pro-inflammatory cytokine transcriptional activation. The expression of E6 and E7 from HPV38 induces the activation and stabilization of PKR and ΔNp73α, thus forming an inhibitory complex with DNMT1 that represses IL-18 gene expression.

In conclusion, in this study, we propose a novel molecular mechanism for the regulation of the IL-18 promoter that involves p53 after UVB irradiation in primary keratinocytes. The presence of β-HPV38 oncoproteins strongly abrogates this mechanism by forming an inhibitory complex DNMT1/PKR/ΔNp73α that represses IL-18 gene expression (Fig. 6). The disruption of this complex leads to a rescue of the basal IL-18 mRNA level. Nevertheless, UVB irradiation does not induce any synergic effect.

In particular, our research provides new possible explanations for the cooperation between UVB irradiation and β-HPV types in cellular transformation. Based on these results, we might hypothesize that virus-mediated p53 dysfunction leads to an aberrant regulation of genes related to inflammasome pathways, enhancing the capacity of the cells to evade immune system control.

However, further studies are necessary to elucidate the mechanisms by which the inhibition of IL-18 expression by HVP38 leads to the development of NMSC.

## MATERIALS AND METHODS

### Cell cultures and treatments

The experiments were carried out in HK isolated from neonatal foreskin and in an HK cell line expressing the hTERT gene in order to prolong the life span of the cells. HK stably expressing HPV38 E6 or E7 as well as HA-ΔNp73α 38HK were generated by retroviral transduction (10, 52). The same donor-derived cell line was used for all the experiments. The 38HK, HA-ΔNp73α 38HK, and hTERT-HK were co-cultured with NIH3T3 feeder layers in fetal bovine serum (FBS)-albumin-dextrose (FAD) medium containing three parts Ham’s F12 medium, one part Dulbecco’s modified Eagle’s medium (DMEM), 5% fetal calf serum (FCS), insulin (5 µg/mL), epidermal growth factor (10 ng/mL), cholera toxin (8.4 ng/mL), adenine (24 µg/mL), and hydrocortisone (0.4 µg/mL). NIKs were co-cultured with J2 feeder layers in FAD medium. Feeder layers were prepared by mitomycin treatment (0.5 mg/mL) for 2 h. NIH 3T3 cells were cultivated in high-glucose (4.5 g/L) DMEM supplemented with 10% FCS. Human keratinocytes isolated from neonatal foreskin (HK), as previously described (53), were provided by Dr. Lamartine J (CNRS, Lyon) and cultured in Keratinocyte Growth Medium 2 (PromoCell).

Cells covered within a thin layer of phosphate-buffered saline (PBS) were exposed to a 50 mJ/cm^2^ dose of UVB radiation using the Bio-Sun UV irradiation system (Vilber Lourmat). After irradiation, the cells were cultured in a humidified chamber for 8 h at 37°C and then harvested.

Transient transfection experiments were performed using Lipofectamine 2000 transfection reagent (Invitrogen) or TransIT-Keratinocytes Transfection Reagent (Mirus) according to the manufacturer’s protocols.

Cells were incubated for 8 h in medium containing cyclic pifithrin-α hydrobromide (PFT) at 20 µM (Sigma); others were incubated for 24 h in medium containing 5-aza-2′-deoxycytidine (Aza) at 30 µM (Sigma).

2AP (Sigma) was prepared in PBS:glacial acetic acid (200:1). Cells were treated for 4 h at 10 mM final concentration; PBS:glacial acetic acid (200:1) was used as a mock-treated control.

### Gene silencing

PKR GeneSolution siRNA was purchased from Qiagen. Briefly, 300 nM siPKR or non-specific siRNA (NSsiRNA) (scramble) was transfected using Lipofectamine 2000 reagent according to the manufacturer’s protocol. Cells were collected after 24 hr.

Sense/antisense oligonucleotides (Table 1) were transfected as previously described (52).

Plasmids for CRISPR/Cas9 were obtained from the Addgene plasmid repository. All single-guide RNAs were designed by Thermo Fisher Scientific. The target sequence information is shown in Table 1. The CRISPR/Cas9 vectors were generated according to the manufacturer’s protocols and then transiently transfected into HK. Purification of the cells carrying the CRISPR/Cas9 vectors was performed 48 h after transfection according to the manufacturer’s protocol (GeneArt CRISPR Nuclease Vector Kit; Life Technologies).

### Reverse transcription and quantitative PCR

Total RNA was extracted using the NucleoSpin RNA II Kit (Macherey-Nagel). The RNA obtained was reverse transcribed to cDNA using the RevertAid H Minus First Strand cDNA Synthesis Kit (Life Technologies) as previously described (33). RT-qPCR was performed using the Mesa Green qPCR MasterMix Plus for SYBR Assay (Eurogentec) with the primers listed in Table 1.

### Immunoblotting

Cells were lysed using IP buffer [20 mM Tris HCl (pH 7.5), 200 mM NaCl, 1 mM EDTA, 0.5% NP-40] supplemented with Complete Protease Inhibitor mixture (Roche). Samples were resolved by SDS-PAGE and transferred to polyvinylidene difluoride membranes (Perkin Elmer). Membranes were blocked in 5% non-fat milk and incubated overnight at 4°C with the appropriate primary antibody. Membranes were probed with the following primary antibodies: β-actin (clone C4; MP Biomedicals), p53 DO-1 (Santa Cruz Biotechnology, sc-126), PKR (Cell Signaling Technology, 3072), phosphorylated PKR Thr446 (Thermo Fischer Scientific, PA5-37704), p73 (Abcam, ab215038), HA-tag (Roche, 3F10), and Lamin B1 (GeneTex, GTX103292).

Images were produced using the Vilber Fusion FX (Vilber) and the ChemiDoc XRS imaging system (Bio-Rad).

### Chromatin immunoprecipitation

ChIP was performed using the Chromatin Shearing Optimization and OneDay ChIP kits (Diagenode) as previously described (54). Briefly, cells were sonicated with Bioruptor Pico (Diagenode) to obtain DNA fragments of 200–500 bp. Sheared chromatin was immunoprecipitated using the indicated antibodies: p53 (DO-1) (Santa Cruz Biotechnology, sc-126), p73 (Abcam, ab215038), HA (Abcam, ab9110), DNMT1 (clone Abnova, 60B1220.1), and phospho PKR T446 (Abcam, ab32036). ChIP-reChIP experiments were carried out as previously described (33). The eluted DNA was used as a template for qPCR. The intergenic region of chromosome 22 was used as a negative control as previously described (55) (Table 1). A negative control region previously described lacking p73 and p53 binding sites was used for ΔNp73α ChIP (56) (Table 1).

### Statistical analysis

Statistical significance was determined using the Student *t*-test with Prism7 (GraphPad). The levels of statistical significance for each experiment (*P ≤ 0.05; **P ≤ 0.01; ***P ≤ 0.001; ****P ≤ 0.0001; ns, not significant) are indicated in the corresponding figures. The error bars in the graphs represent the standard deviations.

## Data Availability

Raw data of all experiments were provided to the editor for review purposes.
